# Supplementary material for: Unravelling the 2e− ORR Activity Induced by Distance Effect on Main-Group Metal InN4 Surface Based on First Principles
Source: Molecules. 2022 Nov 9;27(22):7720. doi: 10.3390/molecules27227720 (PMC9699217; doi:10.3390/molecules27227720)
Supplement: Supplementary file 1 [file molecules-27-07720-s001.zip › molecules-1970742-supplementary.pdf]

Unravelling the 2e- ORR activity induced by distance effect on main-group metal InN4 surface based on first principles

Peng Li [1], Jiawen Xu[2,*] and Yaqiong Su [1,*]

[1] School of Chemistry, Xi'an Key Laboratory of Sustainable Energy Materials Chemistry, State Key Laboratory of Electrical Insulation and Power Equipment, Engineering Research Center of Energy Storage Materials and Devices, Ministry of Education, Xi'an Jiaotong University, Xi'an, 710049, China

[2] General Research Institute of Engineering of Gotion High-Tech, Baohe District, Hefei, 230041, China

* Correspondence: blochstar@163.com; yqsu1989@xjtu.edu.cn

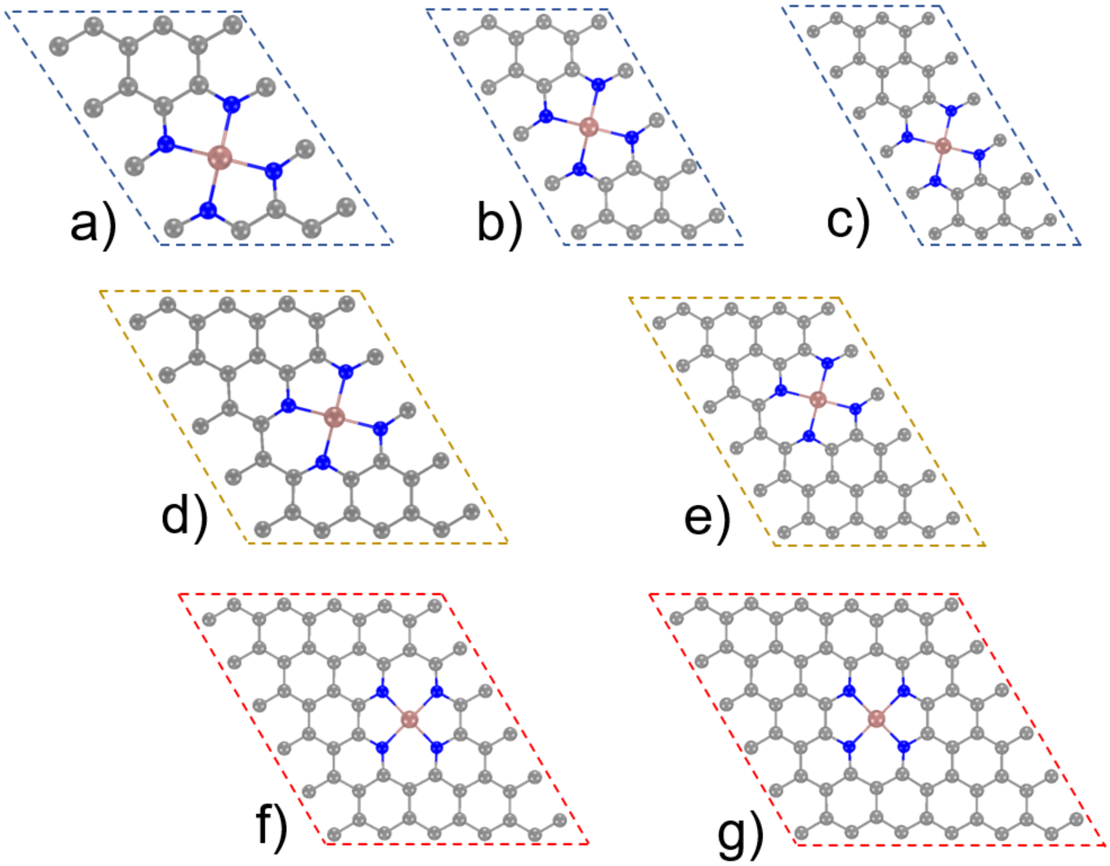

Figure S1 The doping structure of InN4 on the surface of two-dimensional graphene in different unit cells. (**a**) InN_4-3X4; (**b**) InN_4-3X5; (**c**) InN_4-3X6; (**d**) InN_4-4X5; (**e**) InN_4-4X6; (**f**) InN_4-5X6; (**g**) InN_4-6X6; . And gray-black, blue and maroon, are C atoms, N atoms and In atoms, respectively.

Table S1 The doping energy of In atom in different supercells.

	3X3	3X6	4X4	6X6
E_{doping} (eV)	-3.40	-3.53	-3.81	-3.45

Table S2 The adsorption energy of In atom on graphene in different supercells. E_{doping} is defined as $E_{adsorption} = E_{In\text{-}G} - E_{In} - E_{G}$, where $E_{In\text{-}G}$, E_{In} and E_{G} are the energy of the graphene with In atoms adsorbed on its surface (In-G), In atom and graphene, respectively.

	3X3	3X6	4X4	5X5
$E_{adsorption}$ (eV)	-0.96	-1.38	-1.15	-1.34

Table S3 The relative energy of InN_4 calculated by using different convergence threshold.

	cut-off energy (eV)	EDIFF (eV)	EDIFFG	ΔE (eV)
1	400	10^{-4}	-0.05	0
2	400	10^{-5}	-0.02	-0.06
3	400	10^{-7}	-0.01	-0.06
4	450	10^{-4}	-0.05	-0.06
5	500	10^{-4}	-0.05	-0.09

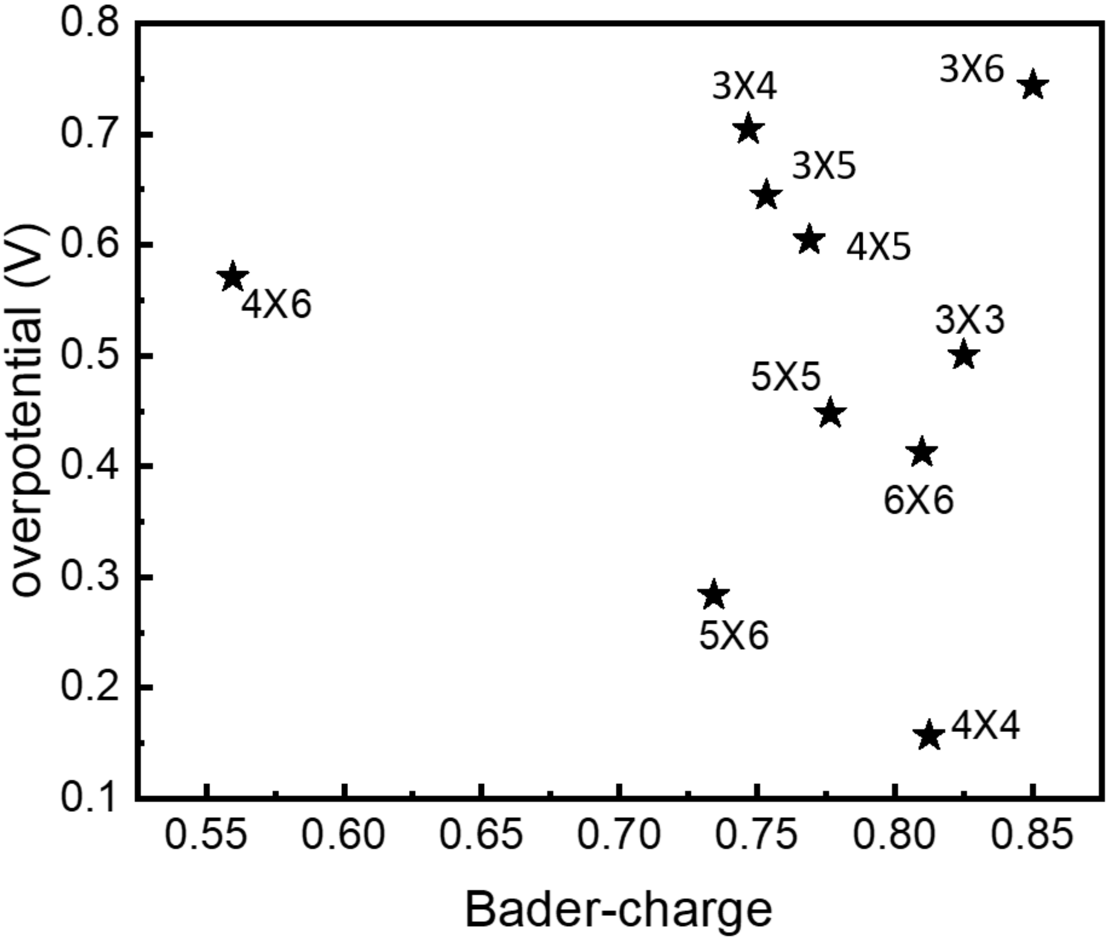

Figure S2 The relationship between 2e⁻ ORR catalytic activity and bader charge.

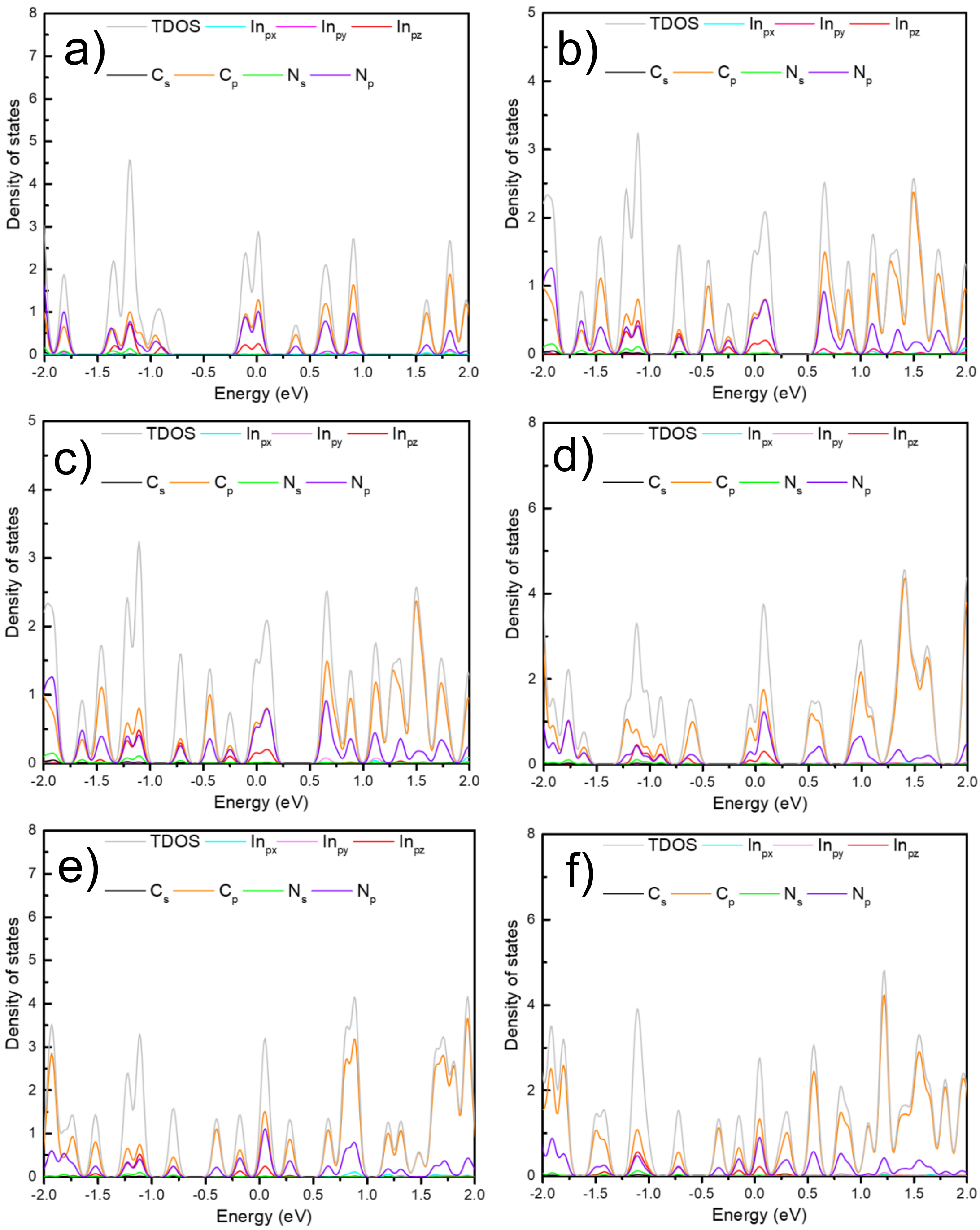
a)
b)
c)
d)
e)
f)
TDOS
In$_{px}$
In$_{py}$
In$_{pz}$
C$_{s}$
C$_{p}$
N$_{s}$
N$_{p}$
Density of states
Energy (eV)

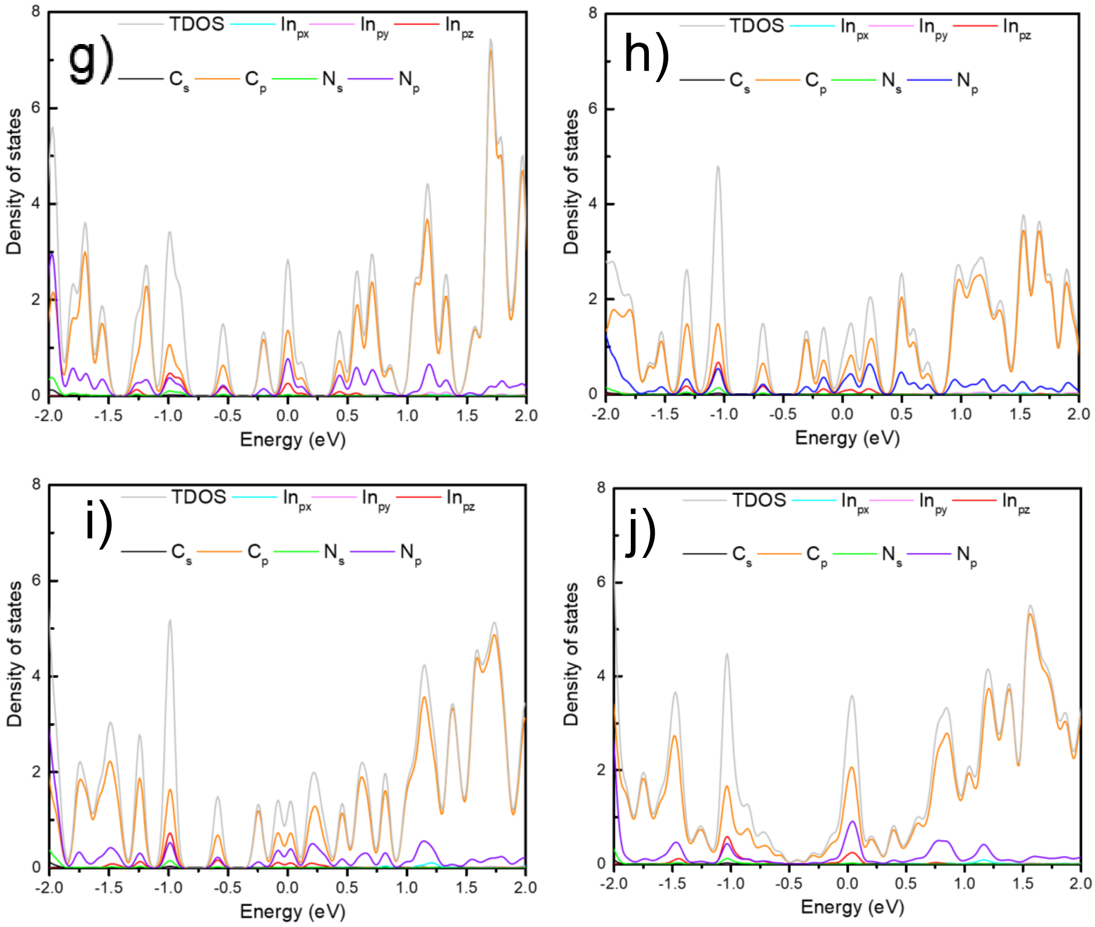

Figure S3 The distribution of density of states (DOS) of InN_4 in different supercells. (**a**) 3X3; (**b**) 3X4; (**c**) 3X5; (**d**) 3X6; (**e**) 4X4; (**f**) 4X5; (**g**) 4X6; (**h**) 5X5; (**i**) 5X6; (**j**) 6X6.

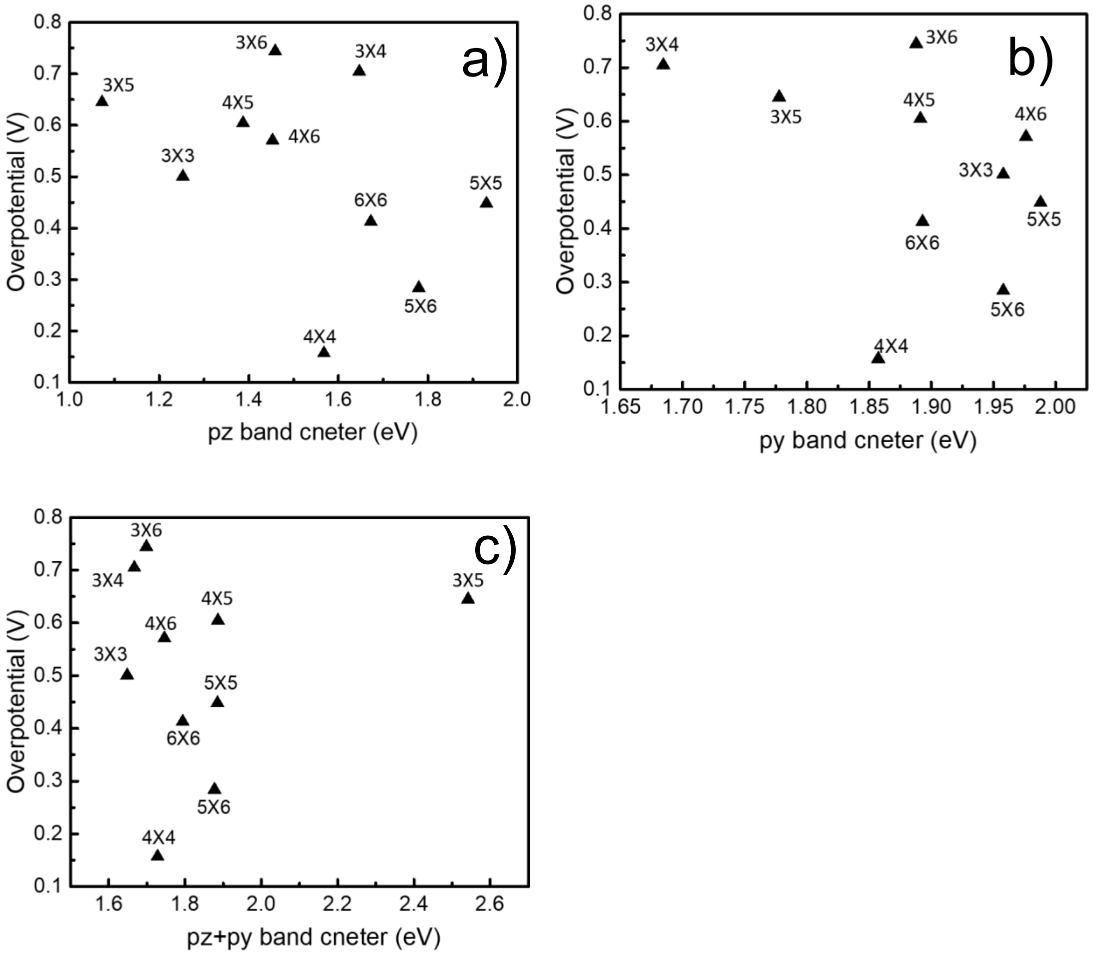

Figure S4 The relationship between band center value and 2e⁻ ORR overpotential. (**a**) pz orbit center; (**b**) py orbit center; (**c**) The center of the superposition of the pz orbital and the py orbital.

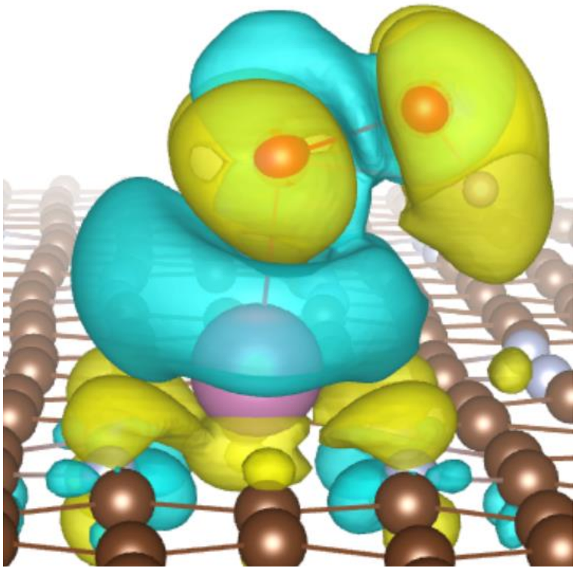

Figure S5 The difference charge distribution of the surface of InN_4 (4X4) with adsorbing OOH. Yellow is the electron accumulation area and cyan is the electron deficient area.

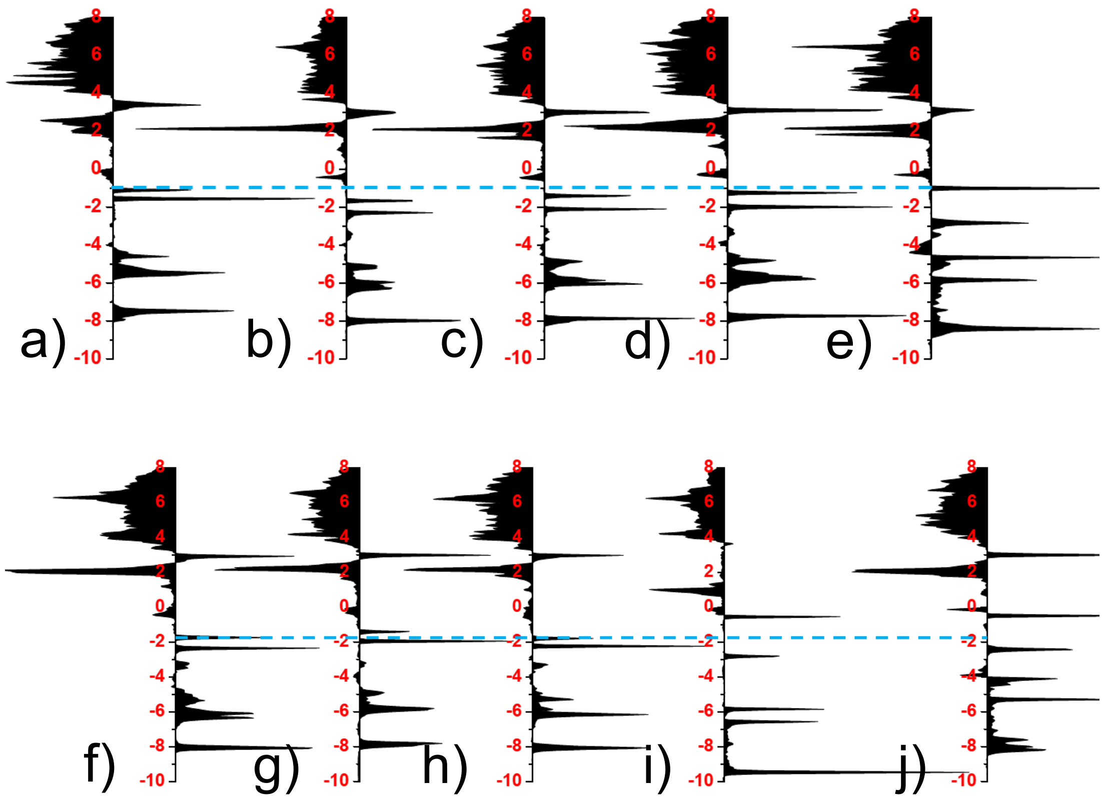

Figure S6 The COHP distribution of In-O bonds in different unit cells of InN_4. The left side is the antibonding component, and the right side is the bonding part (the abscissa is COHP). (**a**) InN_4-3X3; (**b**) InN_4-3X4; (**c**) InN_4-3X5; (**d**) InN_4-3X6; (**e**) InN_4-4X4; (**f**) InN_4-4X5; (**g**) InN_4-4X6; (**h**) InN_4-5X5; (**i**) InN_4-5X6; (**j**) InN_4-6X6.

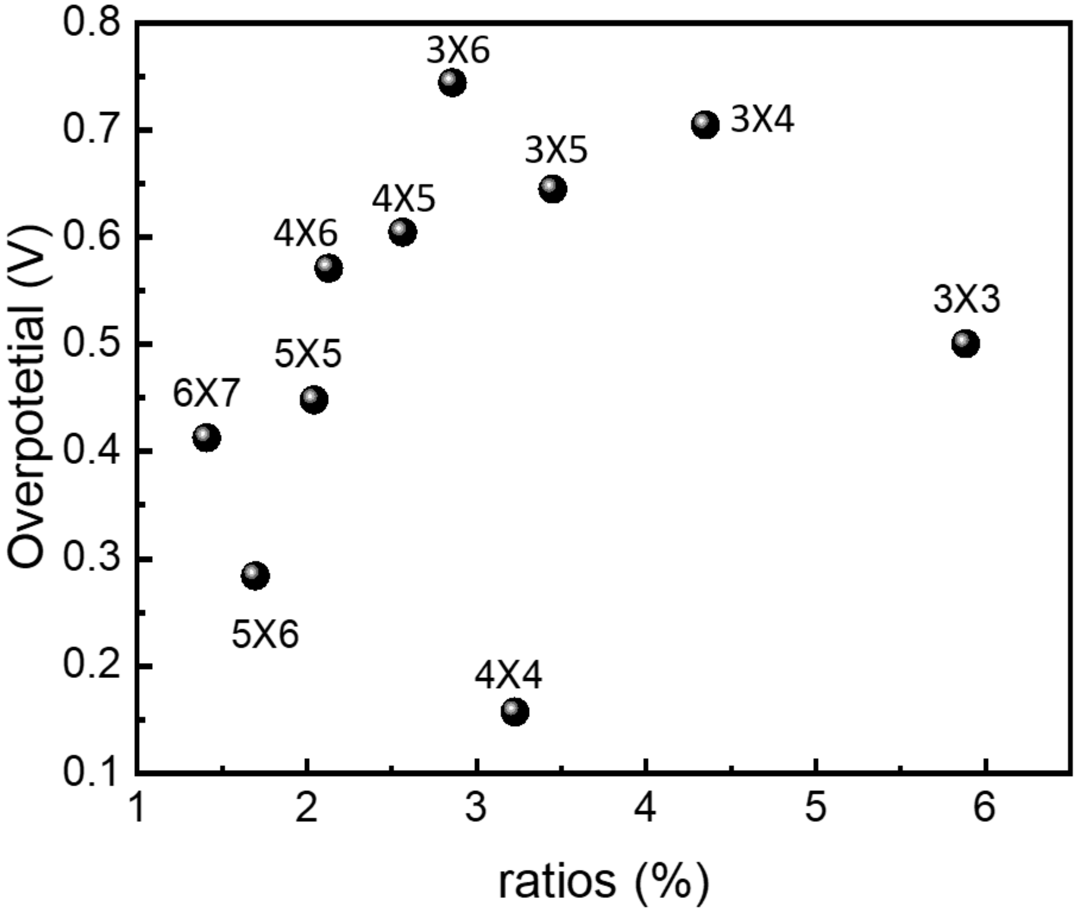

Figure S7 The relationship between the loading ratio of main group metal In atoms and the overpotential of 2e⁻ ORR

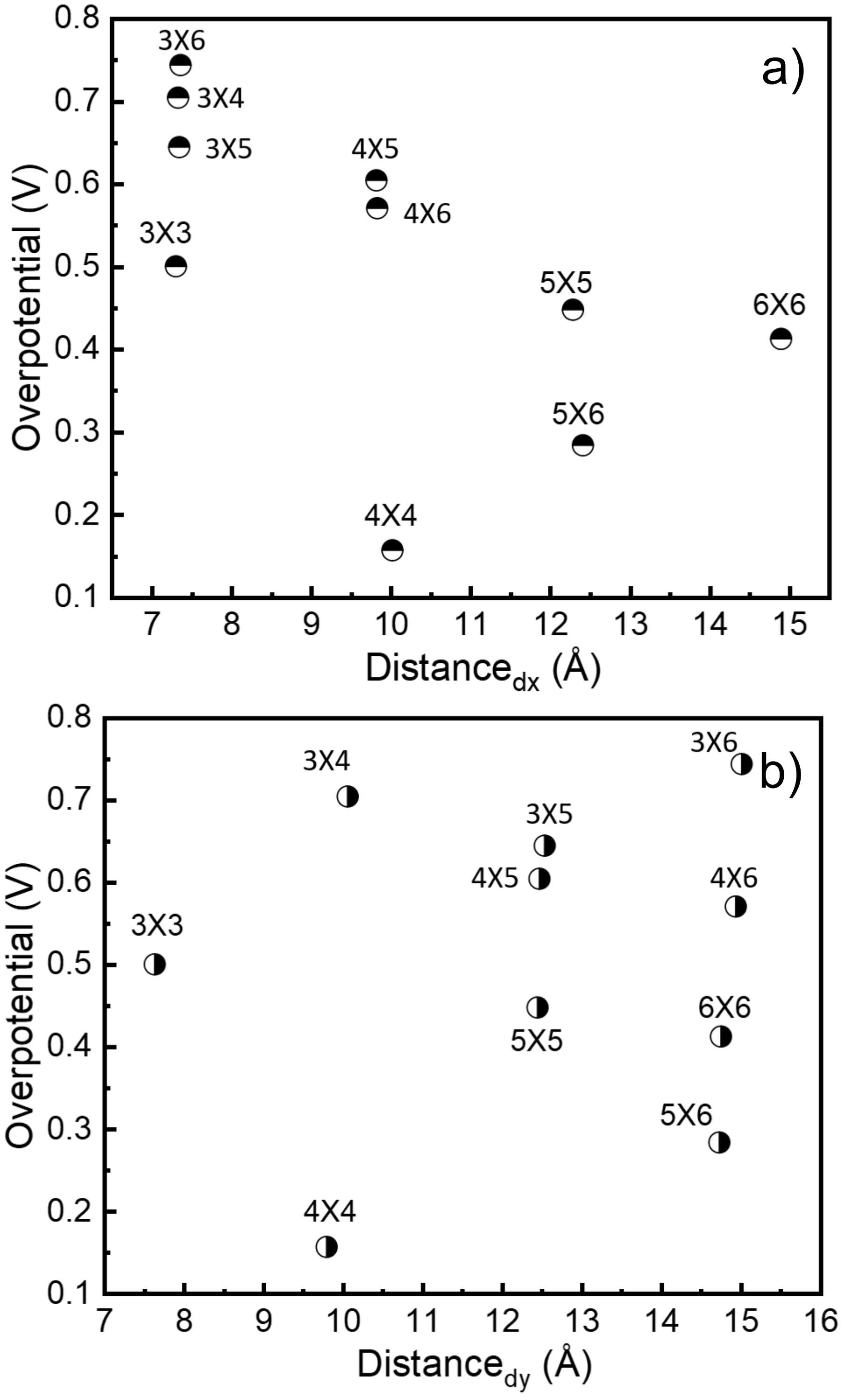

Figure S8 The relationship between the loading ratio of main group metal In atoms and the overpotential of 2e⁻ ORR. (**a**) The spacing of In atoms in the horizontal direction (dx); (**b**) The spacing of In atoms in the vertical direction (dy).

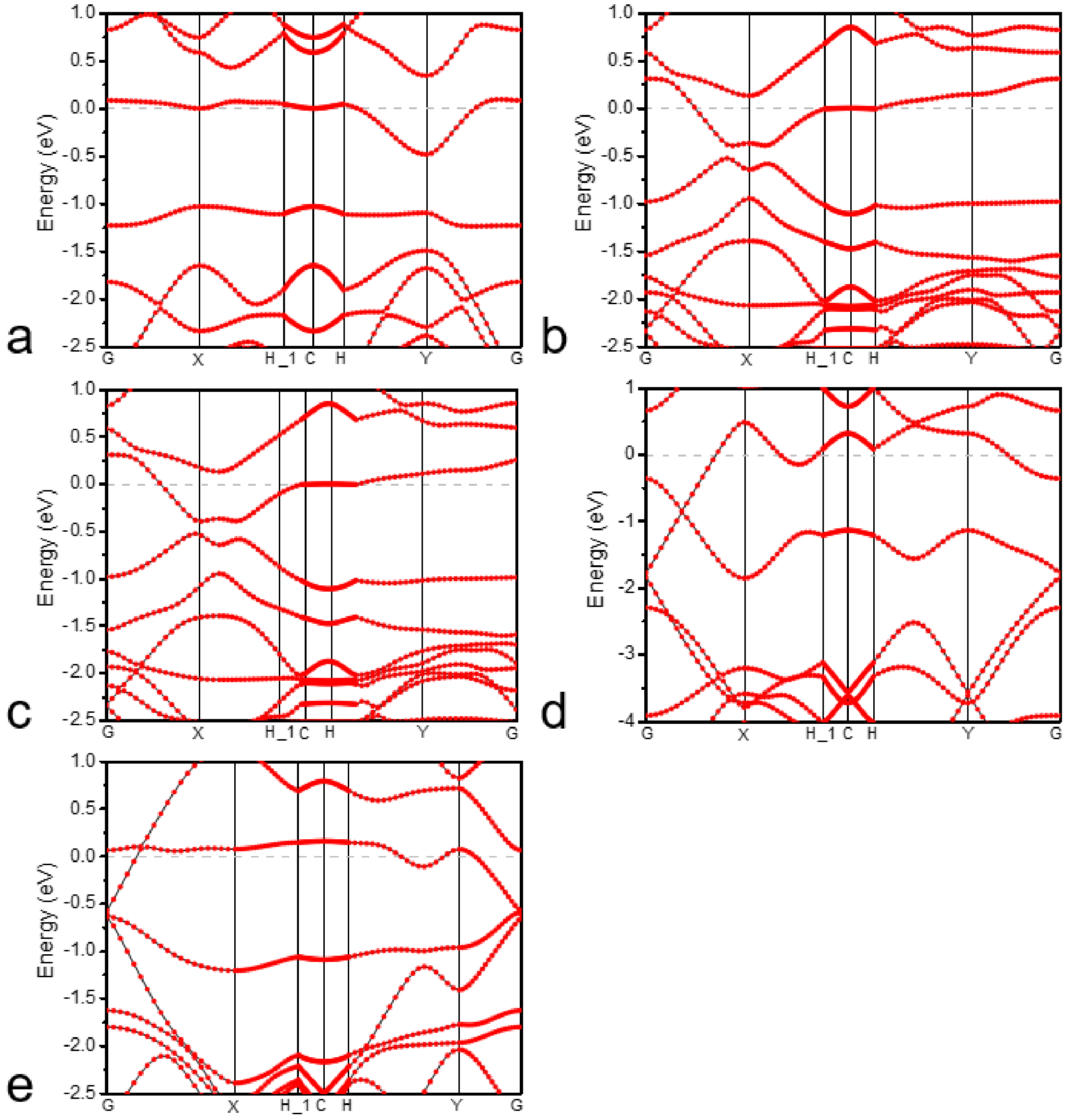

Figure S9 The band structure of InN_4 and the weights of the p_z orbitals in (a) 4X4 (b) 5X6 (c) 6X6 (d) 3X3 (e) 3X6 supercells. The density of the band points reflects the weight of the p_z orbitals.
